# Synthetic Secoisolariciresinol Diglucoside (LGM2605) Prevents Asbestos-Induced Inflammation and Genotoxic Cell Damage in Human Mesothelial Cells

**DOI:** 10.3390/ijms231710085

**Published:** 2022-09-03

**Authors:** Ralph A. Pietrofesa, Shampa Chatterjee, Yuwaraj Kadariya, Joseph R. Testa, Steven M. Albelda, Melpo Christofidou-Solomidou

**Affiliations:** 1 Division of Pulmonary, Allergy, and Critical Care, Department of Medicine, Perelman School of Medicine, University of Pennsylvania, Philadelphia, PA 19104, USA; 2 Institute for Environmental Medicine, Department of Physiology, University of Pennsylvania Perelman School of Medicine, Philadelphia, PA 19104, USA; 3 Cancer Signaling and Epigenetics Program, Fox Chase Cancer Center, Philadelphia, PA 19111, USA

**Keywords:** antioxidant, asbestos, genotoxic cell damage, inflammasome activation, inflammation, LGM2605, malignant transformation, mesothelioma, oxidative stress, secoisolariciresinol diglucoside

## Abstract

Although alveolar macrophages play a critical role in malignant transformation of mesothelial cells following asbestos exposure, inflammatory and oxidative processes continue to occur in the mesothelial cells lining the pleura that may contribute to the carcinogenic process. Malignant transformation of mesothelial cells following asbestos exposure occurs over several decades; however, amelioration of DNA damage, inflammation, and cell injury may impede the carcinogenic process. We have shown in an in vitro model of asbestos-induced macrophage activation that synthetic secoisolariciresinol diglucoside (LGM2605), given preventively, reduced inflammatory cascades and oxidative/nitrosative cell damage. Therefore, it was hypothesized that LGM2605 could also be effective in reducing asbestos-induced activation and the damage of pleural mesothelial cells. LGM2605 treatment (50 µM) of huma n pleural mesothelial cells was initiated 4 h prior to exposure to asbestos (crocidolite, 20 µg/cm^2^). Supernatant and cells were evaluated at 0, 2, 4, and 8 h post asbestos exposure for reactive oxygen species (ROS) generation, DNA damage (oxidized guanine), inflammasome activation (caspase-1 activity) and associated pro-inflammatory cytokine release (IL-1β, IL-18, IL-6, TNFα, and HMGB1), and markers of oxidative stress (malondialdehyde (MDA) and 8-*iso*-prostaglandin F2a (8-*iso*-PGF2α). Asbestos induced a time-dependent ROS increase that was significantly (*p* < 0.0001) reduced (29.4%) by LGM2605 treatment. LGM2605 pretreatment also reduced levels of asbestos-induced DNA damage by 73.6% ± 1.0%. Although levels of inflammasome-activated cytokines, IL-1β and IL-18, reached 29.2 pg/mL ± 0.7 pg/mL and 43.9 pg/mL ± 0.8 pg/mL, respectively, LGM2605 treatment significantly (*p* < 0.0001) reduced cytokine levels comparable to baseline (non-asbestos exposed) values (3.8 pg/mL ± 0.2 pg/mL and 5.4 pg/mL ± 0.2 pg/mL, respectively). Furthermore, levels of IL-6 and TNFα in asbestos-exposed mesothelial cells were high (289.1 pg/mL ± 2.9 pg/mL and 511.3 pg/mL ± 10.2 pg/mL, respectively), while remaining undetectable with LGM2605 pretreatment. HMGB1 (a key inflammatory mediator and initiator of malignant transformation) release was reduced 75.3% ± 0.4% by LGM2605. Levels of MDA and 8-*iso*-PGF2α, markers of oxidative cell injury, were significantly (*p* < 0.001) reduced by 80.5% ± 0.1% and 76.6% ± 0.3%, respectively. LGM2605, given preventively, reduced ROS generation, DNA damage, and inflammasome-activated cytokine release and key inflammatory mediators implicated in asbestos-induced malignant transformation of normal mesothelial cells.

## 1. Introduction

Asbestos refers to a group of naturally occurring hydrated fibrous silicate fibers used in commercial and industrial applications. Preclinical studies in animal exposure models, as well as clinical data, have confirmed that asbestos fiber inhalation can lead to chronic lung conditions, such as pulmonary fibrosis. In addition, asbestos exposure can lead to neoplastic diseases such as malignant mesothelioma (MM) and lung cancer [1,2]. MM is a highly aggressive cancer that arises from the mesothelial cells lining the pleura, pericardium, and peritoneum with a median survival of about one year [3,4,5]. Current therapies, other than surgery in very early disease, are not curative [3]. Presently, MM causes about 3000 deaths per year in the United States and an additional 5000 deaths per year in Western Europe.

Many western countries have restricted asbestos use; however, it is still used in many countries around the world, such as India, Russia, and China. Brazil is the world’s 3rd leading producer of asbestos despite the 2017 ban and, in fact, it was responsible for 96% of asbestos imports into the United States from 2015–2018. Despite an estimated decrease of asbestos mineral consumption from 2 million tons in 2010 to 1 million tons in 2019, a number of asbestos-containing products (roofing tiles, pipes, wall panels, etc.) continue to be manufactured and sold globally. There will, thus, likely be a dramatic increase in MM cases in developing countries, such as India, where the use of asbestos has increased with few precautions taken. However, even in the developed world, important ongoing and persistent exposures still exist. This includes environmental exposures, as occurs in older buildings and schools, and occupational exposures that expose workers to pre-existing asbestos (i.e., plumbers, pipefitters, insulators, insulation removal, and etc.). Furthermore, there are also environmental and domestic exposures from the disturbance of naturally occurring asbestos (NOA). For example, there is an increased risk of MM in areas where mining or asbestos factories have closed. The Libby Asbestos Superfund Site in Libby, Montana, and the BoRit and Ambler Asbestos Piles Superfund Sites in Ambler, Pennsylvania, are classic examples [6].

A major issue in the link between asbestos and cancer is that inhaled asbestos fibers (especially crocidolite asbestos fibers) can persist in the lung for very long periods of time, resulting in continuous damage, even after removal from the exposure. Because of this long latency period (often up to 30–50 years), individuals exposed in the past remain at an increased risk of MM and other cancers throughout their lives, presumably due to continuous, persistent inflammation [7]. Although reduced use of asbestos is likely to reduce the incidence of MM and asbestos-induced lung cancer in the future, it will have no effects on currently exposed populations. In most western countries, workers who have been exposed to asbestos are told to stop smoking to reduce their risk of asbestos-induced lung disease, and are often offered medical surveillance. *There is, thus, an unmet need to develop a chemopreventive agent effective against asbestos-induced MM in high-risk populations*.

Chronic inflammation induced by asbestos exposure is associated with the development and progression of MM. Reducing tissue inflammation and oxidative damage may potentially prevent MM. Currently, it is thought that retained asbestos fibers stimulate both lung phagocytic cells (macrophages) and pleural mesothelial cells to produce reactive oxygen and nitrogen species and inflammatory cytokines that ultimately lead to permanent genetic changes and malignant transformation of the mesothelial cells [8,9,10,11]. Malignant transformation of mesothelial cells following asbestos exposure occurs over several decades; however, amelioration of the molecular signatures of DNA damage, inflammation, and cell injury may impede the carcinogenic process.

Our group has found that flaxseed and its lignan compounds are non-toxic, but effective, multifunctional anti-inflammatory and antioxidant agents that may be useful for lung cancer chemoprevention in cancer cells and a mouse model of chemical carcinogen-induced lung cancer [12,13,14]. We hypothesized that the most bioactive lignan, secoisolariciresinol diglucoside (LGM2605), which is the synthetic version of the natural lignan secoisolariciresinol diglucoside (SDG), could potentially function as a chemopreventive agent for asbestos-induced MM.

Recent studies have shown in an in vitro model of asbestos-induced macrophage activation that LGM2605, given preventively, reduced inflammatory cascades and oxidative/nitrosative cell damage [15]. Although alveolar macrophages play a critical role in malignant transformation of mesothelial cells following asbestos exposure, inflammatory and oxidative processes continue to occur in the mesothelial cells lining the pleura that may contribute to the carcinogenic process. Hillegass et al. have previously shown that exposure to crocidolite asbestos fibers primes and activates the nod-like receptor pyrin domain containing protein 3 (NLRP3) inflammasome in human mesothelial cells [16]. In this study, we asked if LGM2605 might have chemopreventive effects on the other primary cell type affected by asbestos, primary human pleural mesothelial cells, by mitigating cell damage that is associated with malignancy, as we have shown with murine macrophages in previous studies [17,18].

## 2. Results

**Figure 1:**
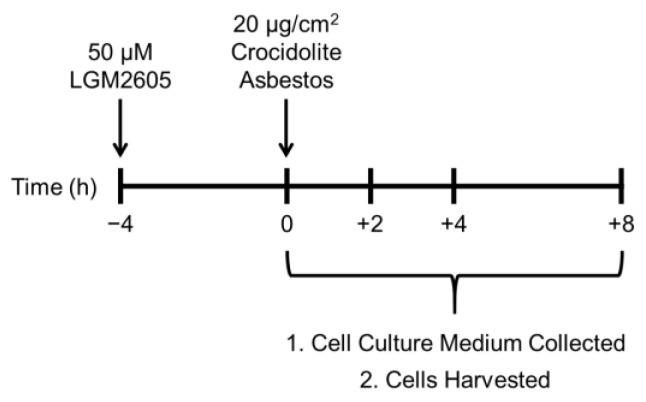
Experimental Plan of Human Pleural Mesothelial Cell Exposure and Harvest Time Points. Human pleural mesothelial cells, derived from a pleural effusion fluid obtained from a single donor, congestive heart failure patient, were plated in a 6-well plate (1 × 10^6^ cells/well) and exposed to 50 µM LGM2605 4 h prior to exposure to sterile crocidolite asbestos fibers (20 µg/cm^2^). Cell culture medium and cells were harvested at 0, 2, 4, and 8 h post asbestos exposure. Cell culture medium was evaluated for ROS release, markers of oxidative and nitrosative stress, proinflammatory cytokine levels, and markers of DNA damage, while cells were evaluated for ROS generation, levels of nuclear factor E2-related factor 2 (Nrf2) and nuclear factor kappa-light-chain-enhancer of activated B cells (NF-κB) transcription factors, caspase-1 activity, levels of γH2AX, and DNA single-strand breaks.

We utilized normal human mesothelial cells isolated from pleural fluid to study the effects of LGM2605 treatment on asbestos-induced ROS generation, oxidative/nitrosative stress, NLRP3 activation, cellular inflammation, and DNA damage. LGM2605 treatment was initiated 4 h prior to exposure to crocidolite asbestos fibers (20 µg/cm^2^), and cell culture medium and mesothelial cells were collected at 0, 2, 4, and 8 h after asbestos exposure (Figure 1).

### 2.1. Asbestos-Induced ROS Generation in Human Pleural Mesothelial Cells Is Reduced by LGM2605 Treatment

**Figure 2:**
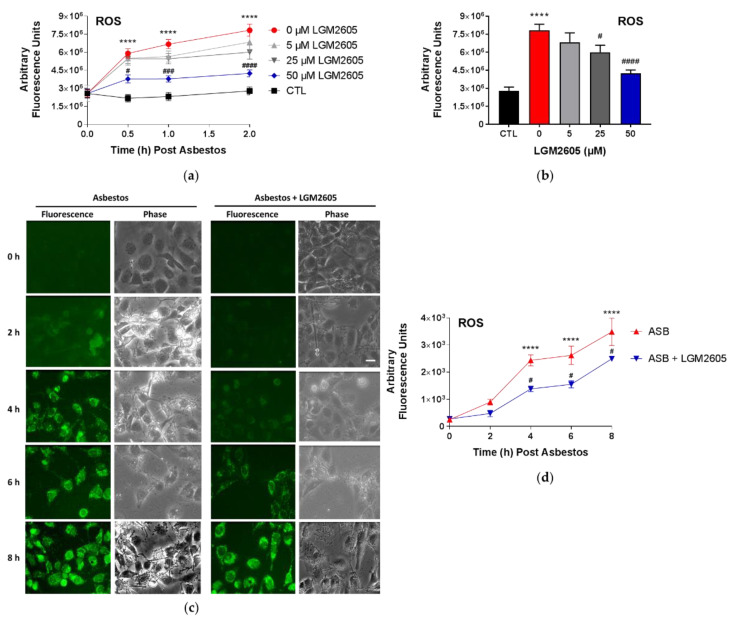
ROS Production Following Asbestos Exposure. Human pleural mesothelial cells were plated in a 96-well plate (2 × 10^4^ cells/well) and exposed to LGM2605 4 h prior to exposure to sterile crocidolite asbestos fibers (20 µg/cm^2^). ROS generation was determined by DCF fluorescence. The dose–response relationship between asbestos-induced ROS generation and various concentrations of LGM2605 treatment (0, 5, 25, and 50 µM) at 0, 0.5, and 2 h post asbestos exposure (**a**). Data are also presented separately for the 2 h time point (**b**). Asbestos-induced ROS generation in mesothelial cells treated with 50 µM LGM2605 was determined through 8 h post asbestos exposure (**c**). Human pleural mesothelial cells were exposed to asbestos and labeled with the oxidative stress sensitive dye, CellROX^®^ Green Reagent. The intensity of the cells in each field (scale bar = 15 μm) was integrated to obtain the total fluorescence intensity of a particular field (**d**). Samples were run undiluted in triplicate and data are presented as mean ± SEM. * indicates a statistically significant difference (*p* < 0.05) from baseline (time 0 h) for cells exposed to asbestos (**** *p* < 0.0001). # indicates a statistically significant difference (*p* < 0.05) between ASB and ASB + LGM2605 treated cells (# *p* < 0.05, ### *p* < 0.001, #### *p* < 0.0001).

We first determined the dose–response relationship between asbestos-induced ROS generation and various concentrations of LGM2605 treatment (0, 5, 25, and 50 µM) at 0, 0.5, and 2 h post asbestos exposure. Exposure to asbestos led to a significant (*p* < 0.0001) increase in DCF fluorescence at 0.5, 1, and 2 h post exposure (Figure 2a). While no significant effect was observed with 5 µM LGM2605, treatment with 25 and 50 µM LGM2605 significantly (*p* < 0.05) reduced ROS generation at 2 h post asbestos by 36.2% and 71.1%, respectively (Figure 2b). Importantly, no significant difference was observed between untreated control (CTL) cells and cells exposed to asbestos and treated with 50 µM LGM2605. As also shown in previous studies [18], 50 µM LGM2605 was sufficient to blunt asbestos-induced ROS generation by human pleural mesothelial cells to levels that were comparable to naïve cells.

We then determined the kinetics of acute asbestos-induced ROS generation in human mesothelial cells following exposure to crocidolite asbestos over an 8 h period using immunofluorescence and the oxidative stress sensitive dye, CellROX™ green reagent (Figure 2c) (Thermo Fisher Scientific, Waltham, MA, USA). Overall, asbestos exposure led to significant (*p* < 0.0001) elevations in ROS generation at 4, 6, and 8 h post asbestos. LGM2605 led to marked reductions in intracellular ROS. The significant (*p* < 0.05) decrease in asbestos-induced ROS generation with 50 µM LGM2605 treatment was observed through 8 h post asbestos exposure (Figure 2d). While levels of intracellular ROS significantly (*p* < 0.0001) increased following asbestos exposure, LGM2605 reduced ROS levels by 43.4%, 40.8%, and 28.5% at 4, 6, and 8 h post asbestos, respectively.

### 2.2. LGM2605 Blunts Cellular Oxidative Stress and Activates Nrf2 following Asbestos Exposure in Human Pleural Mesothelial Cells

Exposure to crocidolite asbestos fibers induces the generation of harmful ROS. We have previously reported on the generation of ROS by peritoneal macrophages undergoing frustrated phagocytosis during the engulfment of rod-like crocidolite asbestos fibers [17]. Here, we examined the effect of exposure to crocidolite asbestos in human pleural mesothelial cells on ROS.

**Figure 3:**
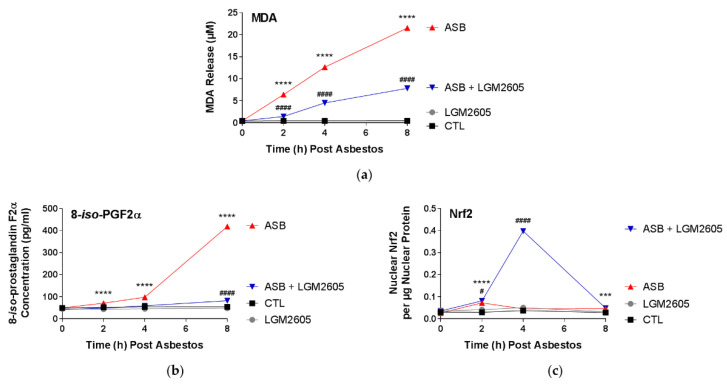
LGM2605 Reduces Markers of Oxidative Cell Damage and Activates Nrf2. Human pleural mesothelial cells were plated in a 6-well plate (1 × 10^6^ cells/well) and exposed to 50 µM LGM2605 4 h prior to exposure to sterile crocidolite asbestos fibers (20 µg/cm^2^). The levels of MDA (**a**) and 8-*iso*-PGF2α (**b**) were determined in the cell culture medium at 0, 2, 4, and 8 h post asbestos exposure. Levels of Nrf2 were determined in cell nuclear extracts (**c**). Data are presented as mean ± SEM. * indicates a statistically significant difference (*p* < 0.05) between ASB and CTL treated cells (*** *p* < 0.001 and **** *p* < 0.0001). # indicates a statistically significant difference (*p* < 0.05) between ASB and ASB + LGM2605 treated cells (# *p* < 0.05 and #### *p* < 0.0001).

MDA release, a marker of lipid peroxidation, was significantly (*p* < 0.0001) elevated following asbestos exposure, from 0.43 µM at baseline to 21.54 µM at 8 h following asbestos exposure. LGM2605 pretreatment (50 µM) significantly (*p* < 0.0001) reduced MDA release by 65.0% at 8 h after asbestos exposure (Figure 3a). 8-*iso*-PGF2α was significantly (*p* < 0.0001) elevated following asbestos exposure from 49.1 pg/mL at baseline to 418.9 pg/mL at 8 h following asbestos exposure. LGM2605 pretreatment (50 µM) significantly (*p* < 0.0001) reduced 8-*iso*-PGF2α release by 92.4% at 8 h after asbestos exposure (Figure 3b). Compared to untreated unexposed (no asbestos) cells, unexposed mesothelial cells treated with 50 µM LGM2605-only had significantly (*p* < 0.05) reduced levels of 8-*iso*-PGF2α release at 2, 4, and 8 h.

We have previously observed an increase in nuclear factor E2-related factor 2 (Nrf2) signaling and endogenous antioxidant enzyme response with LGM2605 treatment (50 µM) in asbestos-exposed peritoneal macrophages [15,17]. We, thus, determined if Nrf2 activation in mesothelial cells may partially account for the observed reductions in markers of oxidative stress. At 2 h post asbestos exposure, levels of nuclear Nrf2 were significantly (*p* < 0.0001) elevated in cells exposed to asbestos (Figure 3c). Pretreatment with LGM2605 (50 µM), led to a statistically significant (*p* < 0.05) further increase in nuclear Nrf2 levels at 2 and 4 h post asbestos exposure, with a 7.86-fold increase relative to asbestos-only exposure at 4 h. Treatment with LGM2605-only (50 µM) led to modest increases in nuclear Nrf2 relative to untreated, unexposed (CTL) mesothelial cells at 2 and 4 h (*p* < 0.01).

### 2.3. LGM2605 Reduces Nitrosative Stress and Nuclear NF-κB in Human Pleural Mesothelial Cells following Asbestos Exposure

**Figure 4:**
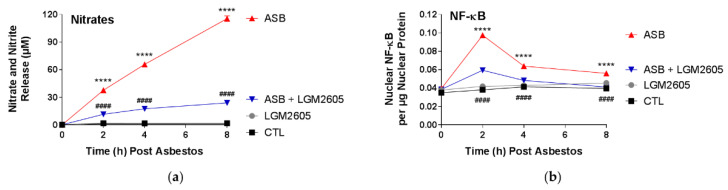
LGM2605 Prevents Asbestos-Induced Nitrosative Cell Damage and NF-κB Activation. Human pleural mesothelial cells were plated in a 6-well plate (1 × 10^6^ cells/well) and exposed to 50 µM LGM2605 4 h prior to exposure to sterile crocidolite asbestos fibers (20 µg/cm^2^). The levels of total nitrates/nitrites (**a**) were determined in the cell culture medium, while levels of NF-κB (**b**) were measured in cell nuclear extracts, at 0, 2, 4, and 8 h post asbestos exposure. Data are presented as mean ± SEM. * indicates a statistically significant difference (*p* < 0.05) between ASB and CTL treated cells (**** *p* < 0.0001). # indicates a statistically significant difference (*p* < 0.05) between ASB and ASB + LGM2605 treated cells (#### *p* < 0.0001).

In addition to harmful ROS, asbestos exposure generates toxic reactive nitrogen species (RNS) that contributes to inflammation and cellular damage [18]. As a marker of nitrosative stress, we determined levels of nitrates and nitrites following asbestos exposure. Total nitrates and nitrites were significantly (*p* < 0.0001) elevated, reaching concentrations of 37.7 µM, 65.8 µM, and 109.5 µM at 2, 4, and 8 h, respectively, following asbestos exposure (Figure 4a). LGM2605 pretreatment (50 µM) significantly (*p* < 0.0001) reduced the concentration of total nitrates and nitrites by 72.9%, 75.2%, and 80.5% at 2, 4, and 8 h post asbestos exposure, respectively. Treatment with LGM2605-only (50 µM) had no significant effect.

Activation of nuclear factor kappa-light-chain-enhancer of activated B cells (NF-κB) is responsible for activation of inducible nitric oxide synthase (iNOS), which leads to the generation of nitric oxide, nitrates, and nitrites. Levels of nuclear NF-κB were significantly (*p* < 0.0001) elevated following asbestos exposure, peaking at 2 h post asbestos (Figure 4b). Treatment with LGM2605 (50 µM) significantly (*p* < 0.0001) decreased levels of nuclear NF-κB by 64.1%, 68.7%, and 90.8% at 2, 4, and 8 h post asbestos exposure, respectively. By 8 h post asbestos exposure, no statistically significant difference was observed between levels of nuclear NF-κB among untreated, unexposed mesothelial cells and cells exposed to asbestos and treated with 50 µM LGM2605.

### 2.4. LGM2605 Prevents NLRP3 Inflammasome Activation and Associated Cytokine Release in Human Pleural Mesothelial Cells

**Figure 5:**
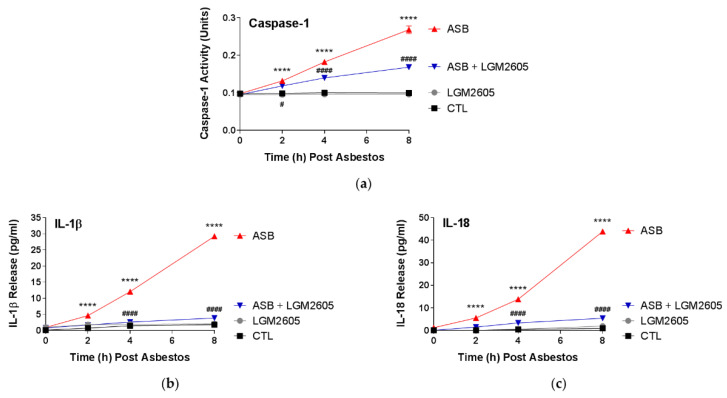
LGM2605 Reduces Asbestos-Induced Caspase-1 Activity and NLRP3 Inflammasome-Activated Cytokine Release in Human Pleural Mesothelial Cells. Human pleural mesothelial cells were plated in a 6-well plate (1 × 10^6^ cells/well) and exposed to 50 µM LGM2605 4 h prior to exposure to sterile crocidolite asbestos fibers (20 µg/cm^2^). NLRP3 inflammasome activation (cleavage and activation of procaspase-1) following asbestos exposure activates IL-1β and IL-18 via activated caspase-1. Caspase-1 activity (**a**) was determined from cell nuclear extracts. The levels of IL-1β (**b**) and IL-18 (**c**) were determined in the cell culture medium at 0, 2, 4, and 8 h post asbestos exposure. Data are presented as mean ± SEM. * indicates a statistically significant difference (*p* < 0.05) between ASB and CTL treated cells (**** *p* < 0.0001). # indicates a statistically significant difference (*p* < 0.05) between ASB and ASB + LGM2605 treated cells (# *p* < 0.05 and #### *p* < 0.0001).

Mesothelial cell exposure to crocidolite asbestos fibers induces the activation of the NLRP3 inflammasome [16]. The activated NLRP3 inflammasome consists of the sensor molecule NLRP3, PYCARD, and active caspase-1, which cleaves the precursor forms of the proinflammatory cytokines, IL-1β and IL-18, to their active forms. Following asbestos exposure, caspase-1 activity increased linearly through 8 h, with fold increases from the baseline of 1.39, 1.79, and 2.67 (Figure 5a). LGM2605 treatment (50 µM) significantly (*p* < 0.05) reduced caspase-1 activity by 51.4%, 46.5%, and 56.5% at 2, 4, and 8 h post asbestos exposure, respectively. Treatment with LGM2605-only (50 µM) had no significant effect.

Levels of IL-1β were significantly (*p* < 0.0001) elevated at 2 h post asbestos exposure (4.60 pg/mL ± 0.30 pg/mL) and continued to increase at 4 h (12.03 pg/mL ± 0.60 pg/mL) and 8 h (29.23 pg/mL ± 0.72 pg/mL) (Figure 5b). 50 µM LGM2605, administered 4 h prior to asbestos exposure, significantly (*p* < 0.0001) reduced IL-1β release by 75.1%, 89.4%, and 92.4% at 2, 4, and 8 h post asbestos exposure, respectively. Levels of IL-18 were also significantly (*p* < 0.0001) elevated at 2 h post asbestos exposure (5.50 pg/mL ± 0.46 pg/mL) and continued to increase at 4 h (13.82 pg/mL ± 0.07 pg/mL) and 8 h (43.90 pg/mL ± 0.79 pg/mL) (Figure 5c). 50 µM LGM2605, administered 4 h prior to asbestos exposure, significantly (*p* < 0.0001) reduced IL-18 release by 73.0%, 78.2%, and 89.6% at 2, 4, and 8 h post asbestos exposure, respectively.

### 2.5. LGM2605 Reduces Proinflammatory Cytokine Release following Asbestos Exposure

**Figure 6:**
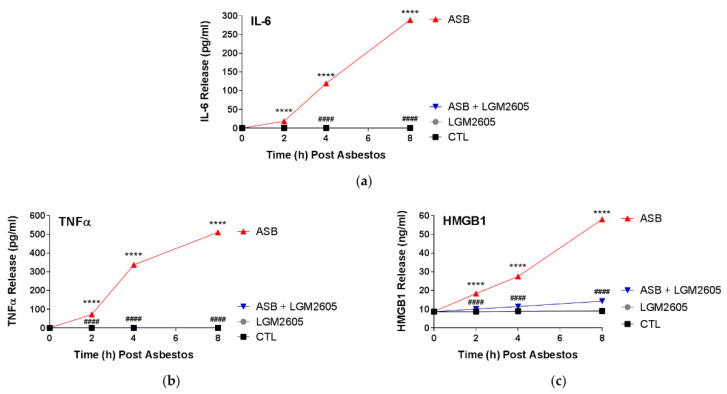
LGM2605 Inhibits Asbestos-Induced Proinflammatory Cytokine Release in Human Mesothelial Cells. Human pleural mesothelial cells were plated in a 6-well plate (1 × 10^6^ cells/well) and exposed to 50 µM LGM2605 4 h prior to exposure to sterile crocidolite asbestos fibers (20 µg/cm^2^). Release of IL-6 (**a**), TNFα (**b**), and HMGB1 (**c**) was determined at 0, 2, 4, and 8 h post asbestos exposure. Samples were run undiluted in triplicate and cytokine concentrations (pg/mL for IL-6 and TNFα, and ng/mL for HMGB1) are presented as mean ± SEM. * indicates a statistically significant difference (*p* < 0.05) between ASB and CTL treated cells (**** *p* < 0.0001). # indicates a statistically significant difference (*p* < 0.05) between ASB and ASB + LGM2605 treated cells (#### *p* < 0.0001).

Asbestos exposure of human mesothelial cells led to significant increases in the release of proinflammatory cytokines: IL-6 (Figure 6a), tumor necrosis factor alpha (TNFα) (Figure 6b), and high-mobility group box 1 (HMGB1) (Figure 6c). Levels of IL-6 were significantly (*p* < 0.0001) elevated at 2 h post asbestos exposure (18.50 pg/mL ± 0.43 pg/mL) and continued to increase at 4 h (119.66 pg/mL ± 2.62 pg/mL) and 8 h (289.05 pg/mL ± 2.94 pg/mL) (Figure 6a). Levels of proinflammatory IL-6 were below the lower limit of detection in human mesothelial cells exposed to asbestos and treated with 50 µM LGM2605 or treated with 50 µM LGM2605-only.

Similarly, levels of TNFα were significantly (*p* < 0.0001) elevated at 2 h post asbestos exposure (72.09 pg/mL ± 0.08 pg/mL) and continued to increase at 4 h (337.13 pg/mL ± 13.44 pg/mL) and 8 h (511.32 pg/mL ± 10.19 pg/mL) (Figure 6b). Levels of proinflammatory TNFα were below the lower limit of detection in human mesothelial cells exposed to asbestos and treated with 50 µM LGM2605 or treated with 50 µM LGM2605-only.

Levels of HMGB1, a critical damage signaling molecule and proinflammatory mediator, were significantly (*p* < 0.0001) elevated at 2 h post asbestos exposure (18.36 ng/mL ± 0.16 ng/mL) and continued to increase at 4 h (27.54 ng/mL ± 0.41 ng/mL) and 8 h (58.09 ng/mL ± 0.02 ng/mL) (Figure 6c). Treatment with LGM2605-only (50 µM) had no significant effect. 50 µM LGM2605, administered 4 h prior to asbestos exposure significantly (*p* < 0.0001) reduced HMGB1 release by 85.2%, 86.9%, and 89.2% at 2, 4, and 8 h post asbestos exposure, respectively.

### 2.6. LGM2605 Treatment Reduces Asbestos-Induced Oxidative DNA Damage in Human Pleural Mesothelial Cells

**Figure 7:**
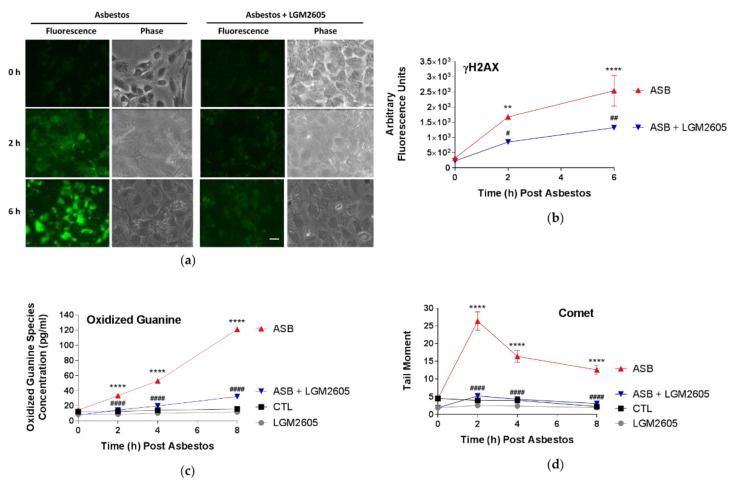
Asbestos-Induced DNA Damage is Ameliorated by LGM2605 Treatment. Human pleural mesothelial cells were plated in a 6-well plate (1 × 10^6^ cells/well) and exposed to 50 µM LGM2605 4 h prior to exposure to sterile crocidolite asbestos fibers (20 µg/cm^2^). Asbestos-induced DNA damage was determined by evaluation of γH2AX, a marker of DNA double-strand breaks, immunostaining (**a**) followed by quantification of the total fluorescence intensity of a particular field (scale bar = 15 μm) (**b**). Additionally, the release of oxidized guanine species (**c**) and the evaluation of DNA single-strand breaks by comet assay (**d**) were determined at 0, 2, 4, and 8 h post asbestos exposure. Samples were run undiluted in triplicate and γH2AX quantification, the release of oxidized guanine species (pg/mL), and tail moment are presented as mean ± SEM. * indicates a statistically significant difference (*p* < 0.05) between ASB and CTL treated cells (** *p* < 0.01, **** *p* < 0.0001). # indicates a statistically significant difference (*p* < 0.05) between ASB and ASB + LGM2605 treated cells (# *p* < 0.05, ## *p* < 0.01, #### *p* < 0.0001).

DNA damage following asbestos exposure was evaluated in mesothelial cells by determining levels of γH2AX (Figure 7a,b), oxidized guanine species (Figure 7c), and DNA single-strand breaks by comet assay (Figure 7c). γH2AX, a marker of DNA double-strand breaks (DSBs), was significantly (*p* < 0.01) elevated at 2 h and 6 h following asbestos exposure (Figure 7a). Treatment with 50 µM LGM2605 reduced the levels of γH2AX by 49.4% and 48.0% at 2 h and 6 h, respectively (Figure 7b). Levels of oxidized guanine species (8-hydroxy-2′-deoxyguanosine, 8-hydroxyguanosine, and 8-hydroxyguanine), indicative of DNA and RNA damage, were also significantly (*p* < 0.0001) elevated at 2 h post asbestos exposure (33.18 pg/mL ± 1.45 pg/mL) and continued to increase at 4 h (52.66 pg/mL ± 0.27 pg/mL) and 8 h (120.98 pg/mL ± 1.56 pg/mL) (Figure 7c). Compared to untreated, unexposed (CTL) cells, mesothelial cells pretreated with LGM2605-only (50 µM) had significantly (*p* < 0.001) reduced levels of oxidized guanine species at 0, 2, 4, and 8 h. 50 µM LGM2605, administered 4 h prior to asbestos exposure, significantly (*p* < 0.0001) reduced the level of oxidized guanine species by 90.9%, 85.3%, and 84.4% at 2, 4, and 8 h post asbestos exposure, respectively.

Asbestos-induced DNA single-strand breaks (SSBs) were determined using the alkaline comet assay. Mean tail moment peaked at 2 h following asbestos exposure and remained significantly (*p* < 0.0001) elevated at 4 and 8 h. LGM2605 pretreatment (50 µM) significantly (*p* < 0.0001) reduced asbestos-induced DNA SSBs by 94.5%, 97.4%, and 91.8% at 2, 4, and 8 h post asbestos exposure, respectively (Figure 7d). While treatment with 50 µM LGM2605-only significantly (*p* < 0.0001) reduced levels of oxidized guanine species as compared to untreated control cells, no significant differences in DNA SSBs were observed.

## 3. Discussion

In this study, LGM2605 treatment (50 µM) of human pleural mesothelial cells was initiated 4 h prior to exposure to asbestos (crocidolite, 20 µg/cm^2^) and supernatant and cells were evaluated at 0, 2, 4, and 8 h post asbestos exposure. Pretreatment with LGM2605 reduced asbestos-induced ROS and DNA damage, as determined by the oxidized guanine species, comet assay analysis, and γH2AX levels. In addition, markers of oxidative and nitrosative stress (CellROX^®^ Green Reagent, MDA, 8-*iso*-PGF2α, and total nitrates and nitrites) were reduced, while promoting activation of Nrf2 signaling. Furthermore, asbestos induced inflammation (NF-κB nuclear translocation and caspase-1 activity) and associated proinflammatory cytokine (IL-1β, IL-18, IL-6, TNFα, and HMGB1) release was ameliorated by the action of LGM2605. Thus, our findings, using a human mesothelial cell asbestos exposure model system, provide novel evidence that LGM2605 may be a good candidate for the chemoprevention of asbestos-induced malignancy.

**Figure 8:**
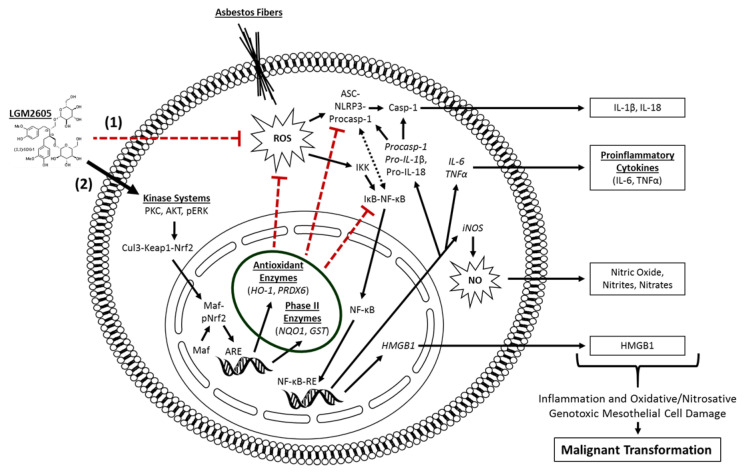
Proposed Mechanism of Asbestos-Induced Mesothelial Inflammation and Oxidative/Nitrosative Cell Damage, and the Protective Effects of LGM2605. Crocidolite asbestos fibers generate harmful ROS/RNS that lead (directly and indirectly) to genotoxic mesothelial cell damage and inflammation through the activation of the NLRP3 inflammasome and NF-κB pathways. LGM2605 reduces mesothelial cell damage and genotoxic stress by (**1**) directly scavenging ROS and (**2**) activating the Nrf2-antioxidant response element (ARE) pathway and upregulating key antioxidant enzymes, such as HO-1 and NQO1. Arrow-headed lines indicate activation and bar-headed lines indicate inhibition. ARE, antioxidant response element; ASC, apoptosis-associated speck-like protein containing a C-terminal caspase recruitment domain; Cul3, cullin-3; GST, glutathione S-transferase; HMGB1, high mobility group box 1; HO-1, heme oxygenase-1; IκB, inhibitor of κB; IκK, IκB kinase; IL-1β, interleukin-1β; IL-18, interleukin-18; IL-6, interleukin-6; iNOS, inducible nitric oxide synthase; Keap1, kelch-like ECH-associated protein 1; LGM2605, synthetic SDG; NQO1, NADPH: quinone oxidoreductase-1; NF-κB, nuclear factor kappa-light-chain-enhancer of activated B cells; NF-κB-RE, NF-κB response element; NLRP3, nod-like receptor pyrin domain containing protein 3; NO, nitric oxide; Nrf2, nuclear factor (erythroid-derived 2)-like 2; ROS, reactive oxygen species; sMaf, small Maf (musculoaponeurotic fibrosarcoma); TNFα, tumor necrosis factor alpha; PRDX6, peroxiredoxin-6.

It is hypothesized that alveolar macrophages and pleural mesothelial cells exposed to asbestos undergo frustrated phagocytosis of elongated fibers; this process is thought to cause chronic production of ROS/RNS and proinflammatory cytokine release, which contribute to DNA damage and subsequent cancer initiation and progression [8]. Our group has extensively studied synthetic SDG (LGM2605) as a potential chemopreventive agent for asbestos-induced diseases [15,17,18,19]. We hypothesized that LGM2605 could act as a chemopreventive agent in an in vitro model of human mesothelial cell exposure to crocidolite asbestos, due to its well-defined anti-inflammatory and antioxidant properties. In the current study, we demonstrate that LGM2605 successfully protects human mesothelial cells from asbestos-induced DNA damage; an effect that has also been previously shown in other primary lung cell types exposed to ionizing radiation [20]. Agents, such as celecoxib, a cyclooxygenase-2 inhibitor, and resveratrol, a botanical from the skin of red grapes, have been evaluated for their antitumor properties using in vitro and in vivo models of malignant mesothelioma [21,22,23]. However, unlike our study, which evaluates cancer prevention and inhibition of cancer initiation in normal mesothelial cells, these investigations are limited to studying cancer progression and potential therapeutics for already-formed MM. In general, there are other compounds that have been investigated regarding their potential usefulness for chemoprevention, with genistein, daidzein, and resveratrol to name a few [24]. However, unlike these compounds, LGM2605 has been shown to exert effects on multiple molecular and cellular targets (scavenging ROS, activating cellular antioxidant defenses, and inhibiting NF-κB- and NLRP3-driven inflammation). Combined, this multipronged action by LGM2605 (see Figure 8) enhances its potential for successful chemopreventive use.

Asbestos-induced genotoxicity, caused by harmful ROS and RNS released by mesothelial cells and macrophages undergoing frustrated phagocytosis of elongated asbestos fibers, may lead to alterations in DNA structure. It has been reported that asbestos fibers alter the chemical structure of DNA, causing chromosomal abnormalities, namely aneuploidy, arising from mitotic errors [25], and DNA double-strand breaks [26]. Although macrophages represent the first responders to asbestos deposition in the lung, mesothelial cells lining the pleural cavity have been implicated in the propagation of inflammation and ROS generation following asbestos exposure. We have previously evaluated the ability of a flaxseed lignan diet, enriched in the SDG, to prevent acute asbestos-induced inflammation and oxidative/nitrosative stress in a murine model of peritoneal asbestos exposure [19]. Synthetic SDG, LGM2605, also prevented asbestos-induced inflammation and oxidative/nitrosative cell damage in elicited murine peritoneal macrophages [17,18]. Similar to our previous findings, we report here a significant inhibition of the proinflammatory cascade in mesothelial cells following asbestos exposure, with marked reductions in NF-κB activation, caspase-1 activity, and NLRP3 inflammasome-activated cytokine (IL-1β and IL-18) release.

Asbestos fibers primarily cause necrotic death to mesothelial cells, whereas apoptotic death is approximately 8–18% [27,28]. Asbestos fibers induce necrotic cell death triggering the release of HMGB1, a nucleus-located protein, to the extracellular milieu [28]. HMGB1 is passively secreted from mesothelial cells exposed to crocidolite asbestos fibers [29]. HMGB1 amplifies the inflammatory response by chemoattracting leukocytes and activating NF-κB, but also serves as an important pro-tumor cytokine that enhances the growth, survival, and invasiveness of pleural mesothelial cells [1]. Downregulating HMGB1 signal seems to have considerable therapeutic significance [30], as HMGB1 leads to the activation of other important molecules that act in an environment prone to carcinogenesis. Once released, HMGB1 binds to the receptor for advanced glycation end products (RAGE) on mesothelial cells and macrophages. HMGB1 release triggers a signaling cascade involving increased levels of TNFα, implicated in the malignant transformation of mesothelial cells [31]. HMGB1 also activates the NLRP3 inflammasome, which, in turn, increases the levels of secreted proinflammatory IL-1β. HMGB1 is an integral part of the danger-associated molecular pattern (DAMP) or alarmin machinery that can activate the innate immune system either alone or in conjunction with proinflammatory cytokines. Therefore, the fact that LGM2605 reduced HMGB1, in both human pleural mesothelial cells and murine peritoneal macrophages, either via reducing its release by stressed cells or by reducing cell death, implies that this agent (LGM2605) can effectively “break” the asbestos-induced signaling cascade that leads to exaggerated inflammation and apoptosis. Frustrated phagocytosis by alveolar macrophages attempting to clear asbestos deposition in the lungs leads to activation of the NLRP3 inflammasome [32]. The NLRP3 inflammasome is implicated in asbestos-induced disease and provides a molecular target for therapeutic intervention. NLRP3 activation also occurs in mesothelial cells and leads to the release of IL-1β [16].

LGM2605 treatment of human mesothelial cells in our experiments showed an inhibitory role of LGM2605 in decreasing TNFα and NF-κB activation. Promotion of cell survival by TNFα occurs through activation of NF-κB, a protein complex responsible for the survival of mesothelial cells following asbestos exposure. In the current study, LGM2605 inhibits NF-κB nuclear translocation and activation of downstream targets, while enhancing activation of Nrf2 and the antioxidant response. In contrast, when DNA damaged cells do not die, but instead proliferate due to the generated NF-κB survival signal, they are susceptible to malignant transformation [31]. The persistence of these conditions, where mesothelial cells undergo cycles of chronic cell death and inflammation, will inevitably lead to the development of MM [33].

Despite the identification of a causal link between asbestos exposure and the development of MM [34], commercial use of asbestos has not ceased, due to its attractive chemical and physical properties, and low cost. It was only in 1987 that the International Agency for Research on Cancer (IARC) recognized asbestos fibers as a group I carcinogen, which led to the eventual ban of asbestos use, with additional attempts at restricting manufacturing, importing, and/or processing, by most countries worldwide. Despite the attempts to restrict asbestos use, environmental and occupational exposures to asbestos persist. Given the lack of effective therapeutics and the poor prognosis for patients with MM, it is disappointing that there is a paucity of clinical studies that have been conducted regarding asbestos-exposed individuals and potential chemopreventive strategies in the last 20 years [2]. Although regulations have been enacted by the Occupational Safety and Health Administration and the United States Environmental Protection Agency to limit use and subsequent exposure to asbestos in the United States since the 1970s, the number of new cases of MM has not changed significantly over time [35]. Moreover, MM mortality has not decreased over time as would be expected; for women, the number of deaths due to MM has increased 25% from 1999 to 2020 (489 deaths in 1999 to 614 in 2020) [36].

As a complete carcinogen, asbestos can act as both an initiator and a promoter. By mitigating the production of asbestos-induced oxidative stress and genotoxic damage, LGM2605 inhibits the ability of asbestos fibers to act as an initiator, and by mitigating asbestos-induced inflammation and release of cytokines, such as IL-1β and TNFα, LGM2605 inhibits the ability of asbestos fibers to act as a promoter. Malignant transformation of mesothelial cells following asbestos exposure occurs over several decades; however, amelioration of the molecular signatures of DNA damage, inflammation, and cell injury may impede the carcinogenic process. Although the idea of treating high-risk individuals with a cancer preventing therapeutic (chemoprevention) sounds simple, several issues need to be addressed for the identification of an appropriate chemopreventive agent. First, the molecular mechanisms by which carcinogens induce cancer are complex [37], outlined by a multistage process, and involving a dynamic interplay between exposures, environments, and genetics; rendering efficacy a challenge and requiring an agent with multiple therapeutic targets/effects. Second, since the agent will be used to prevent or delay carcinogenesis in a large population of healthy, but at-risk individuals (some of whom will never develop the disease), it must be extraordinarily non-toxic, well-tolerated, and affordable [2]. Provided the long latency period from asbestos exposure to MM development and given the ability to identify high-risk individuals with known exposures, there exists an opportunity for chemopreventive intervention.

In the current study, treatment with LGM2605 was initiated 4 h prior to asbestos exposure. While the usefulness of chemopreventive strategies in asbestos-induced MM would most likely be initiated post asbestos exposure, the opportunity for intervention is broad given the long latency from time of exposure to detectable/symptomatic MM. Thus, a safe, non-toxic agent that can be administered during this period may disrupt the carcinogenic process across the entire spectrum of malignant transformation, with the ultimate goal of delaying, or even preventing MM. This is particularly pertinent to asbestos exposure, where persistent asbestos fibers induce continued inflammation and oxidative stress. Additionally, the presence of NAO fibers in communities and the fact that occupations, such as construction workers and firefighters, remain provide opportunities for pretreatment where anticipated asbestos exposures exist.

The current study is strengthened by the use of non-malignant human pleural mesothelial cells, which is the cell type that undergoes malignant transformation leading to MM formation following asbestos exposure. Additionally, our findings of LGM2605 treatment, which has been shown to be safe and non-toxic, of mesothelial cells supports our previously published findings in other in vitro and in vivo models of asbestos exposure. The present data in human mesothelial cells underscore the chemopreventive properties of LGM2605 against asbestos-induced MM formation. Importantly, LGM2605 achieved a downregulation of molecular signatures, such as HMGB1 and IL-1β release, that give rise to the formation of MM following asbestos exposure. These results highlight LGM2605 as a promising chemopreventive agent for the inhibition of human mesothelial cell damage produced by asbestos.

## 4. Materials and Methods

### 4.1. Human Pleural Mesothelial Cells

Mesothelial cells were derived from a pleural effusion fluid obtained from a single donor congestive heart failure patient as previously described [38]. Approval for use of excess pleural fluid was obtained from the University of Pennsylvania School of Medicine Institutional Review Board (Protocol #823659). Human pleural mesothelial cells were plated in 1 mL of cell culture medium (Medium 199: Medium 106 [Thermo Fisher Scientific, Waltham, MA, USA], supplemented with penicillin [100 units/mL] and streptomycin [100 µg/mL], 10 ng/mL epidermal growth factor [EGF], 15% fetal bovine serum [FBS], L-Glutamine [2 mm], and 1 µL/mL of 50 µM hydrocortisone) in a 6-well plate (2 × 10^6^ cells per well) and allowed to adhere to the bottom of the wells for 24 h. They were then used to determine the effects of LGM2605 in preventing crocidolite asbestos-induced generation of ROS and RNS, NLRP3 inflammasome activation, proinflammatory cytokine secretion, and asbestos-induced genotoxicity. Treatment groups were as follows: untreated control (CTL), 50 µM LGM2605-only (LGM2605), 20 µg/cm^2^ asbestos-only (ASB), and 20 µg/cm^2^ asbestos and 50 µM LGM2605 (ASB + LGM2605).

### 4.2. Crocidolite Asbestos (ASB) Exposure

Human pleural mesothelial cells were exposed to sterile UICC crocidolite (SPI Supplies, West Chester, PA, USA) asbestos fibers as previously described [15,17,18]. Briefly, crocidolite asbestos samples were baked overnight, resuspended in 1× phosphate-buffered saline (PBS) at a stock concentration of 800 µg/mL and sonicated for 30 min. The solution of crocidolite asbestos fibers was exposed to ultraviolet light prior to use in cell culture experiments. For all in vitro experiments, human pleural mesothelial cells were exposed to crocidolite asbestos fibers at a concentration of 20 µg/cm^2^ based on our previous studies in murine peritoneal macrophages (see Figure 1) [15,17,18].

### 4.3. LGM2605 Treatment

Chemical synthesis of LGM2605 (synthetic secoisolariciresinol diglucoside) has been previously described [39]. Briefly, secoisolariciresinol diglucosides (*S*,*S*)-SDG (the major isomer in whole grain flaxseed) and (*R*,*R*)-SDG (the minor isomer in whole grain flaxseed) was synthesized from vanillin via secoisolariciresinol and a glucosyl donor (perbenzoyl-protected trichloroacetimidate under the influence of TMSOTf) through a concise route that involved chromatographic separation of diastereomeric diglucoside derivatives. LGM2605 was reconstituted to a stock concentration of 10 mM, and cells were exposed to 50 µM LGM2605 4 h prior to asbestos exposure (see Figure 1). The 50 µM dose of LGM2605 exposure was determined based on previous studies in murine peritoneal macrophages exposed to the same concentration of crocidolite asbestos [15,17,18].

### 4.4. Quantification of Asbestos-Induced ROS Generation

ROS generation following asbestos exposure was determined using the cell-permeant fluorescent probe 2′,7′-dichlorodihydrofluorescein diacetate (H2DCFDA) (Molecular Probes^®^, ThermoFisher Scientific, Waltham, MA, USA) as previously described [17]. Upon cleavage of the acetate groups by intracellular esterases and oxidation, the nonfluorescent H2DCFDA is converted to the highly fluorescent 2′,7′-dichlorofluorescein (DCF). Human pleural mesothelial cells were plated in a 96-well plate (2 × 10^4^ cells/well) and exposed to 20 µg/cm^2^ sterile crocidolite asbestos fibers and treated with LGM2605. Initial studies were conducted to determine the dose–response relationship between asbestos-induced ROS generation and various concentrations of LGM2605 treatment (0 µM, 5 µM, 25 µM, and 50 µM) at 0, 0.5, and 2 h post asbestos exposure. The fluorescence intensity was measured on a SpectraMax i3x Multi-Mode microplate reader (Molecular Devices, Sunnyvale, CA, USA) using an excitation wavelength in the range of 492–495 nm and a fluorescence emission detection at 517–527 nm.

Subsequent studies evaluated the ability of 50 µM LGM2605 to reduce asbestos-induced ROS generation through 8 h post asbestos exposure using CellROX^®^ Green Reagent (Thermo Fisher Scientific, Waltham, MA, USA) as previously described [15]. Human pleural mesothelial cells were treated with LGM2605 (50 µM) 4 h prior to asbestos challenge (20 µg/cm^2^) and evaluated at 0, 2, 4, 6, and 8 h post asbestos exposure. Mesothelial cells were incubated with 5 μM CellROX® Green Reagent (Thermo Fisher Scientific, Waltham, MA, USA) for 20 min at 37 °C after which cells were washed with phenol red-free RPMI and imaged at λ_ex_ 488 nm on a Nikon TMD fluorescence microscope (Nikon Diaphot TMD, Melville, NY, USA) equipped with a Hamamatsu ORCA-100 camera (Hamamatsu Photonics K.K., Hamamatsu City, Japan). All fluorescent cell images were acquired at the same magnification (10× lens), exposure (200 ms exposure), and acquisition settings using the MetaMorph software (Version 7.7, Molecular Devices, Downingtown, PA, USA). The fluorescent images of cells were processed and quantitated for CellROX^®^ Green Reagent fluorescence by the use of ImageJ software (Fiji Version, National Institutes of Health, Bethesda, MD, USA). For each experimental condition, 3–4 fields were imaged for *n* = 3 independent experiments. The intensity of cells in each field was integrated and normalized to area to obtain the total fluorescence intensity, and expressed as arbitrary fluorescence intensity units (AFU). Scale bar = 15 μM.

### 4.5. Evaluation of Lipid Peroxidation

Malondialdehyde (MDA), an indicator of oxidative stress, was measured in the cell culture medium using a commercially available kit (TBARS Assay Kit, Cayman Chemical Company, Ann Arbor, MI, USA) according to the manufacturer’s protocol. Human pleural mesothelial cells were plated in a 6-well plate (2 × 10^6^ cells/well) and exposed to 50 µM synthetic SDG (LGM2605) 4 h prior to exposure to sterile crocidolite asbestos fibers (20 µg/cm^2^). The levels of MDA were determined in the cell culture medium at 0, 2, 4, and 8 h post asbestos exposure. Specifically, levels of thiobarbituric acid reactive substances (TBARS) were quantified by measuring the fluorescence of malondialdehyde-thiobarbituric acid (MDA-TBA) adducts in cell culture medium samples. According to the manufacturer’s instructions, MDA-TBA adducts were formed via acid hydrolysis at 100 °C and measured fluorometrically using a SpectraMax i3x Multi-Mode microplate reader (Molecular Devices, Sunnyvale, CA, USA) with an excitation wavelength of 530 nm and an emission wavelength of 550 nm. Levels of lipid peroxidation in cell culture medium samples are reported as the concentration (µM) of MDA.

### 4.6. Analysis of 8-Iso-Prostaglandin F2a Levels in the Cell Culture Medium

Levels of 8-*iso*-PGF2α, metabolites of tissue phospholipid oxidation, and a biomarker of oxidative stress and antioxidant deficiency, were determined in the cell culture medium using an 8-*iso*-PGF2α enzyme-linked immunosorbent assay (ELISA) kit (Cayman Chemical Company, Ann Arbor, MI, USA) according to the manufacturer’s protocol. Human pleural mesothelial cells were plated in a 6-well plate (2 × 10^6^ cells/well) and exposed to 50 µM synthetic SDG (LGM2605) 4 h prior to exposure to sterile crocidolite asbestos fibers (20 µg/cm^2^). The levels of 8-*iso*-PGF2α were determined in the cell culture medium at 0, 2, 4, and 8 h post asbestos exposure. Cell culture medium samples were run undiluted and the data are reported as the concentration (pg/mL) of 8-*iso*-PGF2α in the cell culture medium.

### 4.7. Analysis of Nitrate/Nitrite Levels in Cell Culture Medium

Levels of total nitrates and nitrites, metabolites of nitric oxide, in the culture medium were determined using a nitrate/nitrite colorimetric assay kit (Cayman Chemical Company, Ann Arbor, MI, USA) according to the manufacturer’s protocol. The assay kit quantifies levels of total nitrates/nitrites (stable breakdown products of nitric oxide) by first converting nitrates to nitrites using nitrate reductase and then measuring total nitrites by adding Greiss Reagent to the reaction mixture, which produces a purple azo compound in the presence of nitrites that can be measured spectrophotometrically. The absorbance of the azo chromophore was measured at 540 nm measured using a SpectraMax i3x Multi-Mode microplate reader (Molecular Devices, Sunnyvale, CA, USA). Cell culture medium samples were run undiluted and the data are reported as the concentration (µM) of total nitrate/nitrites in the cell culture medium.

### 4.8. Nrf2 and NF-κB Transcription Factor Analysis

The presence of nuclear factor E2-related factor 2 (Nrf2) and nuclear factor kappa-light-chain-enhancer of activated B cells (NF-κB) p65 subunit was determined in nuclear extracts isolated from human mesothelial cells exposed to asbestos and harvested at 0, 2, 4, and 8 h post asbestos exposure. Cytoplasmic and nuclear extracts were prepared using a commercially available nuclear extraction kit (Cayman Chemical Company, Ann Arbor, MI, USA). Transcription factor assay kits (Cayman Chemical Company, Ann Arbor, MI, USA) were used to detect nuclear Nrf2 and NF-κB transcription factors, as previously described [17,18]. The transcription factor assay kits utilize a specific double-stranded DNA sequence containing the Nrf2 and NF-κB response element, respectively. The data are reported as the ratio of the absorbance at 450 nm (OD_450_) to the nuclear extract protein concentration (μg). Absorbance measurements were obtained using a SpectraMax i3x Multi-Mode microplate reader (Molecular Devices, Sunnyvale, CA, USA).

### 4.9. Determination of Caspase-1 Activity

Caspase-1 (interleukin-1 converting enzyme, ICE) activity was determined using a commercially available fluorometric assay kit (Caspase-1 Assay Kit, ab39412, Abcam, Waltham, MA, USA), as previously performed [15]. Briefly, the assay kit measures caspase-1 activity from cytosolic extracts utilizing a labeled substrate YVAD-pNA (YVAD is a sequence that is recognized by caspase-1). Cleaved p-nitroanilide (pNA) is then measured fluorometrically using a SpectraMax i3x Multi-Mode microplate reader (Molecular Devices, Sunnyvale, CA, USA) with an excitation wavelength of 400 nm and an emission wavelength of 505 nm. Data are presented as caspase-1 activity (units) based on a caspase-1 calibration curve using recombinant caspase-1.

### 4.10. Quantification of Asbestos-Induced Proinflammatory Cytokine Release

Levels of proinflammatory cytokines, IL-1β, IL-6, IL-18, TNFα, and HMGB1 were determined in the cell culture medium at multiple time points post asbestos exposure (0, 2, 4, and 8 h post asbestos) using ELISA. Samples were run undiluted in triplicate and assays were performed according to manufacturer’s instructions. Levels of IL-1β, IL-6, IL-18, and TNFα are reported as picograms per milliliter (pg/mL) of culture medium, and levels of HMGB1 released into the culture medium are reported as nanograms per milliliter (ng/mL). Human ELISA kits (IL-1β, IL-6, and TNFα) were purchased from BD Biosciences (San Jose, CA, USA), MBL International (Woburn, MA, USA) (human IL-18 ELISA Kit), and Chondrex, Inc. (Woodinville, WA, USA) (HMGB1 Detection Kit). Absorbance measurements were obtained using a SpectraMax i3x Multi-Mode microplate reader (Molecular Devices, Sunnyvale, CA, USA).

### 4.11. γH2AX Immunostaining

Human pleural mesothelial cells were exposed to LGM2605 (50 µM) 4 h prior to asbestos challenge (20 µg/cm^2^), and cells were evaluated at 0, 2, and 6 h post asbestos exposure. Mesothelial cells were fixed, permeabilized, and immunostained using anti-γH2AX (1:200) as primary antibody followed by secondary antibody conjugated to Alexa488 (green). Fluorescence imaging was performed at λ _ex_ 488 nm on a Nikon TMD fluorescence microscope equipped with a Hamamatsu ORCA-100 camera. All fluorescent cell images were acquired at the same magnification (10× lens), exposure (250 ms exposure), and acquisition settings using the MetaMorph software (Version 7.7, Molecular Devices, Downingtown, PA, USA). The fluorescent images of cells were processed and quantitated the use of ImageJ software (Fiji Version, National Institutes of Health, Bethesda, MD, USA). For each experimental condition, 3–4 fields were imaged for *n* = 3 independent experiments. The intensity of cells in each field was integrated and normalized to area to obtain the total fluorescence intensity, and expressed as arbitrary fluorescence intensity units (AFU). Scale bar = 15 μM.

### 4.12. Analysis of Oxidized Guanine Species in the Cell Culture Medium

Levels of markers of oxidative DNA and RNA damage, 8-hydroxy-2′-deoxyguanosine from DNA, 8-hydroxyguanosine from RNA, and 8-hydroxyguanine from either DNA or RNA, were determined in the cell culture medium using an DNA/RNA Oxidative Damage ELISA kit (Cayman Chemical Company, Ann Arbor, MI, USA) according to the manufacturer’s protocol. Human pleural mesothelial cells were plated in a 6-well plate (2 × 10^6^ cells/well) and exposed to 50 µM synthetic SDG (LGM2605) 4 h prior to exposure to sterile crocidolite asbestos fibers (20 µg/cm^2^). The levels of oxidized guanine species were determined in the cell culture medium at 0, 2, 4, and 8 h post asbestos exposure. The absorbance was measured at 410 nm (OD_410_) using a SpectraMax i3x Multi-Mode microplate reader (Molecular Devices, Sunnyvale, CA, USA). Cell culture medium samples were run undiluted and the data are reported as the concentration (pg/mL) of oxidized guanine species in the cell culture medium.

### 4.13. Determination of Asbestos-Induced DNA Damage by Comet Assay

Exponentially growing human pleural mesothelial cells were cultured in 6-well plates and exposed to 50 µM LGM2605 4 h prior to exposure to sterile crocidolite asbestos fibers (20 µg/cm^2^). As previously described [20], cells were processed for Comet assay as per manufacturer’s instructions (Trevigen, Gaithersburg, MD, USA). Briefly, harvested mesothelial cells (1 × 10^5^ cells/mL in 1× PBS) were mixed with LMAgarose^®^ (1:10, *v*/*v*) and immediately pipetted onto CometSlides™. Cells were then lysed (4 °C for 30 min) and kept in the dark for unwinding (RT). Electrophoresis was done in a horizontal electrophoresis unit at 18 volts (200 Amp) for 25 min. Slides were washed twice with distilled water, fixed in 70% ethanol and dried at 45 °C. DNA was stained by SYBR green (Trevigen, Gaithersburg, MD, USA). At least 100 cells were scored per group. Visual analysis of cells and comet tail length was measured using Comet Image Analysis software (Comet Assay IV, Perceptive Instruments Ltd., Haverhill, MA, USA). Images were captured on an Olympus IX51 fluorescence microscope using a monochrome CCD FireWire camera with a 40× objective lens.

### 4.14. Statistical Analysis of the Data

All data were analyzed using two-way analysis of variance (ANOVA) to test for the main effects of time and treatment, and the interaction between these variables, on study outcome measures. If the overall F-test was statistically significant, Tukey’s HSD post hoc tests were conducted to determine significant differences between treatment groups (CTL, LGM2605, ASB, and ASB + LGM2605) within each respective time point. Statistically significant differences were determined using GraphPad Prism version 7.00 for Windows, GraphPad Software, La Jolla, CA, USA, www.graphpad.com. Results are reported as the mean ± the standard error of the mean (SEM) from three separate experiments. Statistically significant differences were determined with *p*-value < 0.05. Asterisks shown in figures indicate significant differences between unexposed, control (CTL) and asbestos-exposed (ASB) groups at each respective time point: * *p* < 0.05, ** *p* < 0.01, *** *p* < 0.001, and **** *p* < 0.0001. A # sign shown in figures indicates significant differences between asbestos-exposed (ASB) and asbestos-exposed and LGM2605-treated (ASB + LGM2605) human pleural mesothelial cells at each respective time point: # *p* < 0.05, ## *p* < 0.01, ### *p* < 0.001, and #### *p* < 0.0001.

## 5. Conclusions

In summary, pretreatment of normal human pleural mesothelial cells with LGM2605 reduced asbestos-induced ROS generation, inflammasome activation and associated cytokine release, and key inflammatory mediators implicated in asbestos-induced malignant transformation of mesothelial cells, while reducing oxidative/nitrosative genotoxic cell damage. We believe that LGM2605 will be an excellent, safe agent for chemopreventive intervention in the context of asbestos exposures.

## Data Availability

Not applicable.
